# A C-peptide complex with albumin and Zn^2+^ increases measurable GLUT1 levels in membranes of human red blood cells

**DOI:** 10.1038/s41598-020-74527-6

**Published:** 2020-10-15

**Authors:** M. Geiger, T. Janes, H. Keshavarz, S. Summers, C. Pinger, D. Fletcher, K. Zinn, M. Tennakoon, A. Karunarathne, D. Spence

**Affiliations:** 1 Department of Biomedical Engineering, Michigan State University, East Lansing, MI 48824 USA; 2 Department of Chemistry, Michigan State University, East Lansing, MI 48824 USA; 3 Institute for Quantitative Health Sciences and Engineering, Michigan State University, East Lansing, MI 48824 USA; 4 Sparrow Hospital, East Lansing, MI 48823 USA; 5 Department of Chemistry and Biochemistry, The University of Toledo, Toledo, OH 43606 USA

**Keywords:** Endocrinology, Diabetes

## Abstract

People with type 1 diabetes (T1D) require exogenous administration of insulin, which stimulates the translocation of the GLUT4 glucose transporter to cell membranes. However, most bloodstream cells contain GLUT1 and are not directly affected by insulin. Here, we report that C-peptide, the 31-amino acid peptide secreted in equal amounts with insulin in vivo, is part of a 3-component complex that affects red blood cell (RBC) membranes. Multiple techniques were used to demonstrate saturable and specific C-peptide binding to RBCs when delivered as part of a complex with albumin. Importantly, when the complex also included Zn^2+^, a significant increase in cell membrane GLUT1 was measured, thus providing a cellular effect similar to insulin, but on a transporter on which insulin has no effect.

## Introduction

It has been nearly 100 years since the first successful administration of insulin to a patient with type 1 diabetes (T1D)^1^. Prior to insulin, the prognosis for people with T1D was poor, with an anticipated lifespan of less than four years following diagnosis, while quality of life was horrible due to a strict adherence to reduced caloric intake, sometimes as low as 400 cal per day preventing proper growth of tissues, bone, and organs^2,3^. Today, people with T1D live within ~ 12 years of the life expectancy of a person without T1D, largely due to the exogenous administration of insulin^4^. However, despite the shrinking gap in average life expectancy, health complications such as kidney failure, nerve damage, vision impairment, and microvascular complications still exist, suggesting a missing therapeutic component that may accompany insulin^5^.

Insulin is secreted from pancreatic β-cells following enzymatic cleavage from the C-peptide hormone. The resultant 51-amino acid length insulin peptide has a well-established role in stimulating the translocation of the GLUT4 glucose transporter to the membranes of skeletal muscle cells and adipocytes, enabling glucose transport through these cells^6^. Interestingly, despite insulin’s ability to lower glucose concentrations in the bloodstream, insulin does not directly affect glucose transport in most cells in the circulation. That is, most bloodstream cells (red blood cells (RBCs), neutrophils, T-cells, B-cells, etc.) do not contain GLUT4 as the primary glucose transporter; specifically, they contain GLUT1, the first of the glucose transporters to be characterized^7^. Despite insulin’s inability to stimulate GLUT1 activity, glucose shuttling through this transporter is crucial for even the most basic of cellular functions, as transport through GLUT1 into cells such as RBCs has been reported to be 50,000 times faster than diffusion alone^8^.

Less understood than insulin is C-peptide, the 31-amino acid length peptide that is better known as the connector of the A and B chains of insulin when part of the proinsulin hormone^9^. C-peptide is secreted in a 1:1 molar ratio with insulin from the pancreatic β-cells into the bloodstream and has a reported half-life in the bloodstream of ~ 30 min, in contrast to insulin’s reported half-life of only a few minutes^9^. Beyond participating in the insulin folding process, researchers have debated for decades about the possibility of C-peptide having biological activity, despite reports in the literature describing improvements in diabetic animal models and some small scale human studies^10–19^. A major reason for skepticism surrounding C-peptide as a biologically active peptide (beyond facilitating insulin folding) is the lack of an identified receptor.

Here, we provide evidence that the elusive C-peptide receptor may not be a receptor for C-peptide alone, but rather an albumin/C-peptide complex. Importantly, when C-peptide is delivered as part of a formulation with albumin that also includes Zn^2+^, the amount of measurable GLUT1 in the RBC membrane is significantly increased. If any of the three components of the formulation are missing, this increase in GLUT1 is not detected. Furthermore, with respect to a downstream physiological effect, the delivery of these components also results in an increase in the release of RBC-derived adenosine triphosphate (ATP), a well-established stimulus of the potent vessel dilator nitric oxide^20^.

## Results

### Albumin binding to RBCs

**Figure 1:**
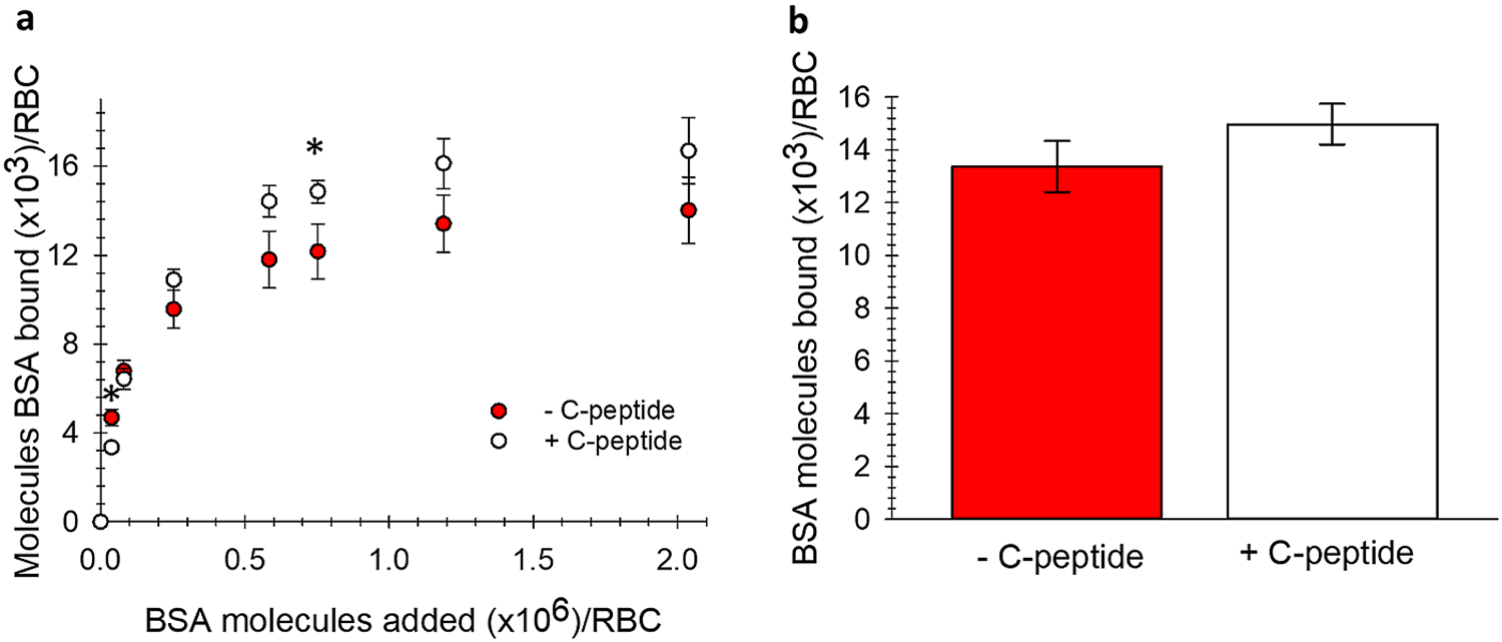
Albumin binding to RBCs. (**A**) The specific binding of BSA without C-peptide (red) in comparison to the specific binding of BSA with C-peptide and Zn^2+^ (white; 20 nM) (n ≥ 4, error = SEM, *p < 0.05). (**B**) The specific binding of BSA without C-peptide (red) in comparison to the specific binding of BSA with C-peptide (white; 100 nM). The experiments in (**B**) were conducted on the same day to compare the difference in BSA binding on the same donor cells. The difference between with and without C-peptide is significantly different than zero (p < 0.01). It is important to note that without albumin, C-peptide binding to RBCs does not occur (n = 5, error = SEM).

Determination of albumin binding to RBCs was facilitated using technetium-labeled bovine serum albumin (BSA-^99m^Tc; Note: While there is a difference in albumins, previously unpublished data from our lab has demonstrated that there is not a significant difference in RBC C-peptide uptake between bovine and human albumins). An ultrafiltration experiment was conducted to demonstrate that BSA-^99m^Tc was able to carry C-peptide in a similar manner as the unlabeled BSA (Supplementary Figure 1)^21^. Increasing concentrations (ranging from 0 to 2700 nM, or ~ 2.0 × 10^6^ molecules) of BSA-^99m^Tc were added to RBCs prepared in an albumin-free physiological salt solution (AF-PSS). To serve as a blocking experiment, a second sample set was prepared by adding increasing amounts of the BSA-^99m^Tc to human RBCs that were prepared in albumin-containing physiological salt solution (PSS). These blocking experiments (shown in Supplementary Figure 2), when subtracted from the AF-PSS binding, result in a specific binding curve that can be used to quantitatively determine the number of albumin molecules that are specifically bound to the RBC. The generation of such a specific binding curve was repeated in the presence of C-peptide as a binding ligand to the labeled albumin. The data in Fig. 1a is an overlay of the specific binding curves of BSA-^99m^Tc in the presence and absence of C-peptide. The BSA-^99m^Tc specifically binds to RBCs in the absence of a binding ligand (in this case, C-peptide) and saturates at an average of 14,021 (± 1489) BSA molecules/RBC with an equilibrium dissociation constant of 1.14 (± 0.07) × 10^–7^ M and a B_max_ of 1.94 (± 0.02) × 10^–8^ M, or approximately 13,900 receptor molecules/RBC. In continuance, Fig. 1a also shows that BSA-^99m^Tc specific binding saturates at an average of 16,695 (± 1479) BSA molecules/RBC when C-peptide and Zn^2+^ are present. The resulting equilibrium dissociation constant is 2.00 (± 0.05) × 10^–7^ M and the B_max_ is 2.50 (± 0.01) × 10^–8^ M, or approximately 17,900 receptor molecules/RBC.

The experiments shown in Fig. 1a were conducted on different days from different blood donors; to improve experimental rigor, RBCs were prepared in 2700 nM BSA-^99m^Tc, with and without C-peptide, following a blood draw from a single donor and prepared on the same day. In addition, each sample was prepared in AF-PSS to demonstrate total binding, and in PSS (which contains BSA) to show non-specific binding, as performed for the saturation experiments that were shown in Fig. 1a. These experiments enabled a direct comparison of BSA specific binding with or without C-peptide on RBCs drawn from a single donor and measured on the same day. The results from these studies, shown in Fig. 1b, revealed an average increase of 1606 (± 492) BSA molecules/RBC when BSA and C-peptide were added to the cells relative to BSA alone.

**Figure 2:**
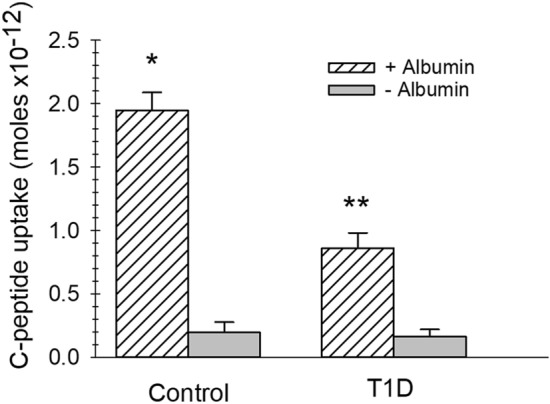
C-peptide binding to control and T1D RBCs. C-peptide binding to control and T1D RBC when 20 nM C-peptide was added to RBCs in the presence (striped bars) and absence (gray bars) of albumin (n ≥ 7, error = SEM, *p < 0.05 to control RBC no albumin. **p < 0.05 to T1D RBC, no albumin.).

The increase in BSA specific binding with C-peptide was just under 2700 BSA molecules/RBCs when extracted from the binding curve data in Fig. 1a and just over 1600 when compared directly using a single donor’s RBCs (Fig. 1b). Either way, these data suggest a specific binding site for BSA, as well as a separate site for BSA that is bound to C-peptide and Zn^2+^. To add further evidence to this hypothesis, we measured the amount of C-peptide binding to RBCs (using ELISA for C-peptide) in the absence and presence of BSA. Figure 2 shows the difference in C-peptide molecules binding to each RBC is 1.7 pmol (or ~ 1800 molecules) greater when in the presence of BSA, a value that is statistically equal to the difference in BSA-^99m^Tc binding when C-peptide is present (shown in Fig. 1). We also measured the amount of C-peptide bound to the RBCs from people with T1D, and while the amount bound is significantly lower than that of the control RBCs, it is still significantly increased from any binding that would occur in vivo (because people with T1D typically have non-detectable levels of C-peptide). Both control and T1D RBCs bind significantly less C-peptide in the absence of albumin. When added with albumin and C-peptide, the amount of bound Zn^2+^ to the RBCs follows the same pattern and is shown in Supplementary Figure 3.

### RBC GLUT1 content

**Figure 3:**
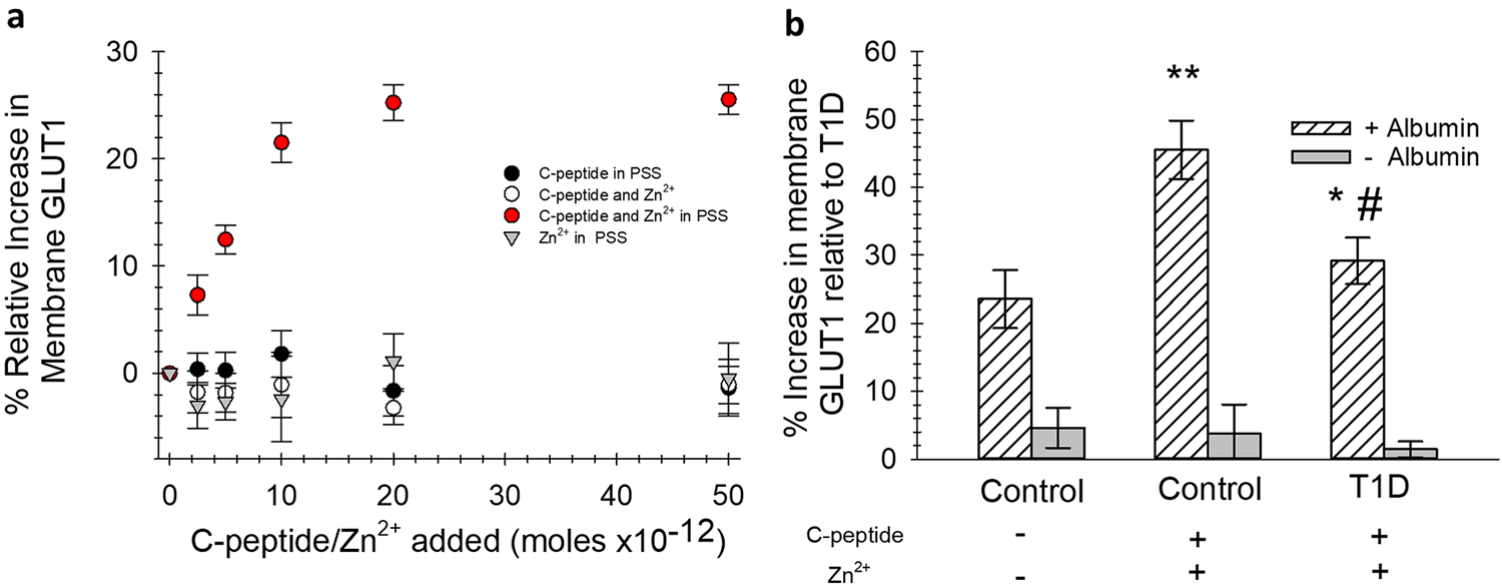
Monitoring GLUT1 levels in RBC membranes. (**A**) The increase in GLUT1 levels in membranes after the addition of varying concentrations of C-peptide in the presence or absence of both Zn^2+^ or albumin. All increases are reported relative to basal levels of the GLUT1 in control donor cells in an albumin-containing buffer (PSS). The increase in red circles is for RBCs with varying concentrations of C-peptide added in the presence of both PSS and Zn^2+^. Black circles show samples containing C-peptide in PSS alone (no Zn^2+^), whereas gray triangles represent samples containing Zn^2+^ alone in PSS (with no C-peptide). White circles represent samples containing C-peptide and Zn^2+^ in an albumin-free form of the PSS (AF-PSS), in other words no albumin was present. A relative increase in measurable RBC membrane GLUT1 is only measured in the presence of albumin/C-peptide/Zn^2+^ (n ≥ 5, error = SEM). (**B**) The increase in measurable membrane GLUT1 for RBCs obtained from healthy control donors and donors with T1D, all realtive to basal levels of GLUT1 determined in membranes of the T1D cells and in the presence (striped bars) and absence (gray bars) of albumin (n ≥ 7, error = SEM, **p < 0.05 to Control RBC, *p < 0.05 to T1D RBC, #p > 0.1 to Control RBC).

The GLUT1 and β-1 spectrin (housekeeping protein) content in RBC ghosts were measured by sodium dodecyl sulfate polyacrylamide gel electrophoresis (SDS–PAGE) and western blot analysis (as shown in Supplementary Figure 4). The GLUT1 band thickness was normalized to the β-1 spectrin band thickness for each sample. The GLUT1 to spectrin ratio was then measured against a control sample ran on the same gel. The percentage increase in GLUT1 to spectrin ratio was then determined in RBC ghosts where the intact RBCs had been subjected to varying concentrations of C-peptide while in the presence and absence of albumin and Zn^2+^. The results in Fig. 3a reveal a striking resemblance to the albumin and C-peptide binding curves in Fig. 1a, especially with respect to the concentration of C-peptide at which the amount of measurable GLUT1 in the cell membrane no longer increases. Furthermore, in the absence of albumin, C-peptide, or Zn^2+^, there is no increase in measurable membrane GLUT1.

The data in Fig. 3b show the increase in measurable GLUT1 levels upon addition of 1 mL of 20 nM C-peptide and Zn^2+^, in the presence and absence of albumin, to RBCs obtained from healthy controls and people with T1D. All increases are relative to the basal GLUT1 to spectrin ratio of the T1D samples. The control samples contained 23.5% (± 4.5%) more basal level of GLUT1 than the T1D samples. The membrane GLUT1 in the control samples increased when in the presence of the C-peptide and Zn^2+^ to 45.5% (± 3.3%). Importantly, the measurable GLUT1 in the membranes of RBCs from T1D donors subjected to C-peptide and Zn^2+^ increased to 29.2% (± 3.4%), a value that is statistically equal to the basal levels of the control RBC GLUT1 levels.

### RBC ATP release

**Figure 4:**
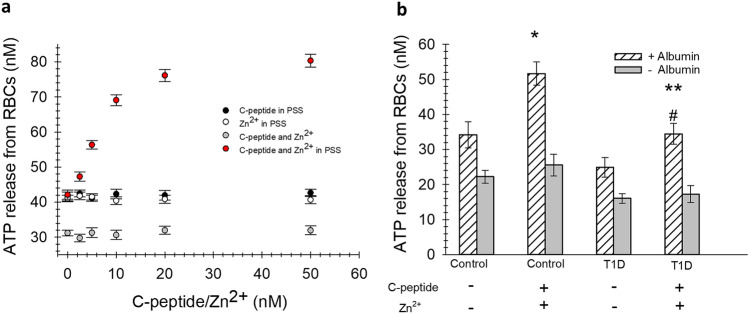
Changes in RBC-derived ATP. (**A**) The RBC-derived ATP increases with added C-peptide in the presence of albumin and Zn^2+^ (red circles). There is no significant increase in ATP release from the control RBCs in samples containing C-peptide in PSS (but no Zn^2+^, black circles) or cells containing Zn^2+^ in PSS (but no C-peptide, white circles). RBC-derived ATP was lowest for cells in AF-PSS (gray circles), even in the presence of C-peptide and Zn^2+^ (n ≥ 6, error = SEM). (**B**) Changes in RBC-Derived ATP occur only in the presence of Zn^2+^, C peptide, and albumin. Neither RBCs obtained from control donors, nor T1D donors were affected by C-peptide/Zn^2+^ in AF-PSS (gray bars). However, ATP release by both types of RBCs increased when Zn^2+^, C-peptide, and albumin are present (striped bars) ([Zn^2+^] = 20 nM, [CP] = 20 nM, n ≥ 7, error = SEM, *p < 0.05 to Control RBC, **p < 0.05 to T1D RBC, #p > 0.4 to Control RBC.).

RBC-derived ATP was measured using the luciferin/luciferase assay with chemiluminescence detection to determine if functional aspects of the albumin/C-peptide/Zn^2+^ binding to the cell follow a similar pattern as that shown for binding studies in Figs. 1 and 3. The basal levels of extracellular ATP (non-lysed cells, 0 nM levels of added C-peptide/Zn^2+^) are shown in Fig. 4a. The average ATP release from RBCs in an albumin-free buffer is 31.2 (± 0.80) nM, while the ATP present from RBCs in an albumin-containing buffer was 41.6 (± 1.3) nM. However, as the concentration of C-peptide and Zn^2+^ added to the RBCs is increased, the ATP release increases from 42.1 (± 1.3) nM to 76.1 (± 1.5) nM. When these same concentrations are added to RBCs in an albumin-free buffer, the ATP release remains statistically unchanged. It should be noted that ATP release begins to plateau at the same points where the amount of C-peptide and Zn^2+^ show saturation, approximately 20 pmol of each added. In a separate study using control and T1D donors, we investigated the ATP release from the RBCs (different from those used to obtain the curve in Fig. 3a) in the presence and absence of C-peptide and Zn^2+^ with and without albumin. The ATP release from control and T1D RBCs treated with C-peptide and Zn^2+^ increased significantly in comparison to a separate aliquot obtained from the same cells that were not treated with C-peptide and Zn^2+^. Importantly, this increase was not detected when the additions were made to an albumin-free buffer.

## Discussion

Discovered just over 5 decades ago, C-peptide, the 31-amino acid length peptide that connects the A and B chains of insulin and is part of the hexameric proinsulin hormone, has long been considered by most scientists to be an inactive biological molecule^9^. However, a surge of reports in the early 1990s stimulated a renewed interest in C-peptide as a possible missing component alongside insulin replacement therapy. Numerous reports describe beneficial effects of exogenous C-peptide replacement therapy in studies using rat models of T1D and in humans with T1D^11,14,22–29^. There are also many reports involving C-peptide’s beneficial effects on cells, in vitro^10,13,16,18,30,31^. Unfortunately, there have been many obstacles to C-peptide being used as a replacement strategy in a clinical setting. For example, most of the studies involving human subjects have been small scale projects, and a recent clinical trial using a pegylated form of C-peptide as a potential therapeutic for diabetic neuropathy didn’t make it past phase IIb of the trial^15^. However, perhaps the main obstacle in using C-peptide as any sort of auxiliary therapy in T1D is the lack of an identified receptor.

There have been reports suggesting that C-peptide binds to a G-protein coupled receptor on the surface of multiple cell types^32,33^. In our previous studies, we have only been able to measure C-peptide binding to cells when in the presence of albumin, although the amount of albumin binding to the cells had never been determined. Prior reports stated that albumin specifically binds to red blood cells^34^. The results in Fig. 1 demonstrate a specific and saturable binding of albumin to the RBC, and the amount of albumin binding to the RBC increases when C-peptide is in the solution with albumin. Interestingly, the increase in albumin molecules bound to the RBC is statistically equivalent to the molecules of C-peptide that bind (as determined by ELISA) to the RBC^35^. In the absence of albumin, there is no significant measurable amounts of C-peptide bound to the cell^35^. These results suggest that the missing receptor for C-peptide is not necessarily a receptor for just C-peptide, but rather a complex formed between albumin and C-peptide, especially considering albumin’s ability to carry proteins and peptides in vivo and the concept of receptors that are specific for albumin-ligand complexes^34^.

Albumin binding would also potentially explain C-peptide’s ~ 30-min half-life in the circulation^9^. It is this longer half-life that enables C-peptide to serve as a biomarker for diagnosing T1D, as insulin’s half-life in the circulation is only 2–3 min^9^. In fact, insulin’s role in the circulation is sometimes not completely understood; while insulin’s ability to lower blood glucose levels is undisputed (whether secreted from the pancreatic β-cell granules or administered exogenously), its blood lowering ability is not due to a direct effect on bloodstream cells. Previous research from our group has demonstrated that insulin has no effect on the measurements (C-peptide uptake, ATP release, etc.) involving RBCs^36,37^. Most cells in the bloodstream (*e.g*., RBCs, neutrophils, T-cells, B-cells, macrophages, etc.) are primarily GLUT1 containing cells^7^. In contrast, insulin stimulates glucose uptake into cells through the GLUT4 glucose transporter, found on such cells as adipocytes and skeletal muscle cells^38^. One of the key findings in the work presented here is the ability of an albumin/C-peptide/Zn^2+^ complex to increase the levels of measurable GLUT1 in the RBC membrane. We use the term “measurable” rather than “translocated” GLUT1 in the membrane because we did not quantitatively determine any GLUT1 moving from the cytosol to the cell membrane. However, when RBCs were treated with the complex, we were able to detect increases in the amount of GLUT1 in the membrane that could be labeled by an antibody during western blot analysis. Further evidence of GLUT1 already present in the membrane prior to treatment is shown in Supplementary Figure 5 where fluorescence microscopy of Chinese hamster ovary (CHO) cells transfected with a green fluorescent protein (eGFP)-labeled GLUT1 are clearly clustered in the cell membrane; in contrast, mCherry-labeled GLUT4 in the same cells are more clustered in the cytosol. These findings are in agreement with a recent report that GLUT1 is already present in the membranes of certain cells but becomes active upon disaggregation^39^. Importantly, many research laboratories have reported that the addition of C-peptide to cells creates a more deformable and less rigid membrane, a feature that could be important to RBCs obtained from people with T1D who have very little, if any, C-peptide in their circulation, due to the destruction of the pancreatic β-cells and have been reported to have RBCs that are more rigid than healthy controls^18,30,40–43^.

The improvement of RBC deformability and GLUT1 activity could have downstream beneficial effects. It is well-established that people with T1D have increased risk of such complications as neuropathy, retinopathy, nephropathy, and cardiovascular disease, all of which are related to microvascular blood flow^5^. Regardless of the mechanism for its release from the RBC, nitric oxide is a well-established vessel dilator and determinant of blood flow. The RBC has been shown to directly participate in the production of blood flow by direct release of nitric oxide or by the release of ATP. Our data in Fig. 4 show that the basal level of ATP derived from the RBCs of people with T1D is lower than that of the control RBCs, but upon addition of the albumin/C-peptide/Zn^2+^ complex, the release increases to a value that is statistically equal to that of the control RBCs. This increase could be increased even further in vivo where it has been shown that RBC-derived ATP also is increased during times of shear-induced deformation and hypoxia, both of which depend on a deformable cell to trigger mechano-induced signaling pathways that lead to ATP release^44–47^.

## Conclusion

People with T1D administer exogenous insulin due to destruction of pancreatic β-cells that produce insulin in vivo. C-peptide, once connected to the insulin as the proinsulin hormone, is released from the β-cells in a 1:1 molar ratio with insulin. However, C-peptide is not administered exogenously as an auxiliary therapy with insulin for several reasons, including the lack of an identified receptor and unsuccessful large-scale trials involving human subjects. Here, we provide evidence that the elusive C-peptide receptor may actually be a receptor for an albumin/C-peptide/Zn^2+^ complex which, when bound to the RBC, stimulates the activation of the GLUT1 transporter, a transporter that is not affected by insulin. This binding has many potential beneficial downstream effects including an improvement of cell deformability and the release of a known stimulus (ATP) of the potent vasodilator and determinant of blood flow, nitric oxide. The results presented here will hopefully stimulate new investigations to seek out the protein that is binding to the albumin/C-peptide/Zn^2+^ complex, determine enhancements in glucose clearance through GLUT1 in the RBC and other GLUT1 containing cells, as well as its in vivo effects on diabetes-related complications.

## Methods

### Isolation and purification of RBCs

All research involving human subjects was approved by the Biomedical and Health Institutional Review Board (BIRB) at Michigan State University and all studies and procedures were performed in accordance with the relevant guidelines and regulations set forth by the BIRB. Signed informed consent was obtained from all participants and/or their legal guardians prior to participation in the study. Whole blood was obtained from consenting donors through venipuncture and collected into heparinized tubes (Fisher Scientific, Waltham, MA)^35^. The whole blood was then centrifuged at 500×*g* for 10 min, and the plasma and buffy coat were subsequently removed via aspiration. The remaining RBCs were resuspended in a physiological salt solution (PSS), containing 4.7 mM KCl (Fisher Scientific), 2.0 mM CaCl_2_ (Fisher Scientific), 140.5 mM NaCl (Sigma Aldrich, St. Louis, MO), 12.0 mM MgSO_4_ (Fisher Scientific), 21.0 mM tris(hydroxymethyl) aminomethane (Invitrogen, Carlsbad, CA), 5.5 mM dextrose (Sigma Aldrich) and 0.5% bovine serum albumin (BSA; Sigma Aldrich; purity ≥ 99% as shown in Supplementary Figure 6) at pH 7.40^35^. An albumin-free version of the PSS (AF-PSS) was also used in these studies, comprised of all the PSS reagents except for the albumin. The RBCs are centrifuged again, the buffer is aspirated off, and new buffer is added for a total of three washes. The RBC hematocrit is determined utilizing a StatSpin MP microhematocrit centrifuge (Beckman Coulter, Brea, CA) and digital hematocrit reader (StatSpin CritSpin Beckman Coulter) for 7% RBC samples to be prepared. RBCs were prepared the day of the experiment and used within 8 h of collection.

### Radiolabeling bovine serum albumin

BSA was incubated with 99.6 nmol succinimidyl 6-hydrazinonicotinate (HYNIC; courtesy of Dr. Gary Bridger, AnorMED, Inc., Langley, British Columbia, Canada) in dimethylformamide for one hour where the conjugation occurs through the 1′ amine groups on the BSA. The BSA-HYNIC was placed into a Slide-A-Lyzer 10 K MWCO dialysis cassette (Thermo Scientific, Rockfort, IL) to remove excess HYNIC. The dialysis cassette was placed into phosphate buffered saline (PBS; 10.1 mM Na_2_HPO_4_ (Sigma Aldirch), 2.7 mM KCl, 136.9 mM NaCl, 1.8 mM KH_2_PO_4_ (Sigma Aldrich) at pH 7.40) at 4 °C for 2 h before the PBS was replaced with new PBS and left overnight at 4 °C. The following morning, sodium pertechnetate (TcO_4_; Cardinal Health, Swartz Creek, MI) was incubated with a previously prepared 0.25 mM tin chloride (Acros Organics, Geel, Belgium) /0.21 M tricine (Sigma Aldrich) kit for 15 min. The tin reduced the TcO_4_^-^ and the tricine acted as a coligand reagent to stabilization the complex with HYNIC^48^. A portion of this solution (150 μL) was then added to the BSA-HYNIC complex and incubated for an additional 30 min. After incubation, 1 mL of the solution was added to a 10 mL 6 K Pierce polyacrylamide desalting column (Thermo Scientific) to separate the free ^99m^Tc from the BSA-^99m^Tc. The column was washed with 1 mL PBS eight times and the activity of each collected fraction was read utilizing a CRC-25R dose calibrator (Capintec Inc., Florham Park, NJ). The first fraction with the highest activity was tested for free or colloidal ^99m^Tc by thin layer chromatography (TLC) using Tec-Control ^99m^Tc chromatography strips (Biodex, Shirley, NY). The fraction was dotted (2 μL) onto two Tec-control dark green chromatography strips and placed into either PBS or methyl ethyl ketone (MEK; Acros Organics). The TLC strip was size dependent as the smaller ^99m^Tc travels up the strip faster than the labeled BSA, therefore, the top portion of the strip can be cut off and both portions analyzed using a 2480 WIZARD^2^ automatic gamma counter (Perkin Elmer, Waltham, MA). The TLC strip in PBS provides the percentage of free ^99m^Tc, and the strip in MEK provides the percentage of colloidal ^99m^Tc, as only TcO_4_^-^ travels up the MEK solvent front^49^. In addition, a Lowry assay was completed for the selected fraction to determine the concentration of BSA.

### Radiolabeling sample preparation

Once the concentration of BSA was determined through a Lowry assay, samples could be created with the desired amount of BSA-^99m^Tc, and the samples were incubated for 2 h at 37 °C. Half of the samples were created with excess unlabeled BSA to block BSA-^99m^Tc binding, which demonstrates non-specific binding, while the other half were created without excess unlabeled BSA to demonstrate total binding. In this way, the specific binding of BSA to RBCs could be calculated by subtracting the non-specific binding of BSA from the total binding of BSA. In addition, BSA binding to the RBCs was analyzed with or without C-peptide (Peptide 2.0; purified by HPLC) or Zn^2+^ (20 nM; C.C.I., Vernon, CA). After incubation, cells were centrifuged (750×*g* for 1 min) to allow for the supernatant to be removed, and AF-PSS was added. Cells were centrifuged again and washed five times with AF-PSS to remove loosely adsorbed proteins. Samples were then measured using a 2480 WIZARD^2^ automatic gamma counter.

### Calculations and data analysis

Sample counts per minute (CPM) from the gamma counter were converted to micrograms through a calibration curve and later to BSA molecules/RBC. Values, such as K_d_ and B_max_, were obtained utilizing SigmaPlot 13.0 nonlinear regression global curve fitting. Analysis of means was performed using a one tailed t-test: two-sample assuming equal variances. A p value < 0.05 was considered to be significant.

### C-peptide uptake by RBCs

In order to measure C-peptide uptake by both T1D and healthy control RBCs, in various matrices, samples were prepared with a 7% hematocrit and 20 nM C-peptide in buffer with and without albumin. Following isolation and purification of the RBCs, the RBCs were incubated in each buffer. The RBCs that were to be incubated in the albumin-free buffer were prepared by washing in albumin-free buffer. The RBCs for all other samples were washed in buffer containing albumin. Samples were prepared in their respective buffers and allowed to incubate at 37 °C for 2 h. Following incubation, the samples were centrifuged, and the supernatant was removed and saved for analysis with commercially available C-peptide ELISA kits (ALPCO, Salem, NH). It is important to note that the amount of C-peptide on the RBCs was calculated by subtracting the amount left in the supernatant from the known amount added.

### Preparing RBC ghost samples for GLUT1 analysis

Three sets of samples were prepared in regular PSS containing C-peptide only, Zn^2+^ only, and a Zn^2+^ and C-peptide mixture. The last set of samples contained Zn^2+^ and C-peptide in AF-PSS. Zn^2+^ and C-peptide concentrations, when present, were 0, 2.5, 5, 10, 20, and 50 nM. After a 2-h incubation at 37 °C and centrifugation, the supernatant was removed, and the sample tubes were filled with 1 mL lysis buffer (10 mM Tris HCl (Invitrogen) and 0.2 mM EDTA (J.T. Baker, Phillipsburg,NY) at a pH of 7.2). The samples were stored at 4 °C for 30 min and then centrifuged at 22,000×*g* for 15 min at 4 °C. The supernatant was then removed, and the pellet was resuspended in the lysis buffer. Once again, the tubes were centrifuged at 22,000×*g* and 4 °C for 5 min, the supernatant was removed, and the RBCs were resuspended in lysis buffer. This process was repeated three times total, or until no more hemoglobin was visible (red color). The lysis buffer was then removed, and the pellets were stored at − 20 °C until use.

### GLUT1 SDS–PAGE and western blot analysis

SDS–PAGE and subsequent western blot was used to probe GLUT1 concentrations. A 10% polyacrylamide gel was used, and the gel was subject to 80 V for 20 min and then 120 V until the buffer reached the bottom of the gel by visual inspection, or about 90 min. Once the proteins were separated on the gel, all proteins were transferred to a polyvinylidene difluoride (PVDF) transfer membrane (EMD Millipore, Burlington, MA) at 15 V overnight for further western blot analysis.

Prior to the addition of antibodies to the membrane, the membrane was blocked for 1 h in 5% dry milk in tris-buffered saline with tween-20 (TBST; Sigma Aldrich). After washing, primary antibodies for GLUT1 (Abcam, Cambridge, MA) and β-1 spectrin (Novus Biological, Littleton, CO; both diluted 1:1000; 15 μL in 15 mL of tris buffered saline (TBS) and 0.75 g dry milk), were added to the PVDF membrane and allowed to incubate for 1 h at room temperature. Once rinsed, anti-rabbit and anti-mouse secondary antibodies (Sigma Aldrich; diluted 1:3000; 5 μL in 15 mL of TBST and 0.75 g dry milk) were added and allowed to incubate for one hour. Sequential incubation with nitro-blue tetrazolium chloride (NBT; Sigma Aldrich) and 5-bromo-4-chloro-3′-indolyphosphate p-toluidine salt (BCIP; Sigma Aldrich) produced a colorimetric indication of the GLUT1 and β-1 spectrin present, and the data was analyzed by scanning the membrane to a computer and utilizing ImageJ software to quantify band thickness and intensity. GLUT1 band thickness was normalized to β-1 spectrin band thickness, and all samples were normalized to the control samples.

### Detection of RBC released ATP

Luciferin/luciferase chemiluminescence assay was utilized to quantify the amount of ATP released by RBCs. Samples were prepared as described in the GLUT1 sample preparation section, with and without C-peptide, Zn^2+^, and albumin. Samples were incubated at 37 °C for 3 h. The standard addition method was used for the quantification of ATP release by preparing increasing concentrations of ATP standards in the corresponding buffer (PSS or AF-PSS). An ATP stock (Sigma Aldrich) was prepared by adding 1.32 g of ATP to 10 mL of water followed by serial dilutions in PSS to prepare 20 μM and 2 μM solutions. Next, using the 2 μM stock solution in PSS, 1 mL of the final concentrations of ATP standards were prepared. Standards for the albumin-free samples were prepared in AF-PSS.

Four aliquots (120 μL) of each RBC sample were added to a black 96-well plate (Corning, Corning, NY) and 30 μL of ATP standard solutions of increasing concentrations were added to the RBC aliquots. Lastly, 10 μL of luciferin/luciferase (prepared by dissolving 5 mg of luciferin (Gold Biotechnology, St. Louis, MO) and 100 mg firefly lantern extract (Sigma Aldrich) in 5 mL deionized water) were added to each sample. The chemiluminescence signal of the samples were measured using a FlexStation 3 spectrophotometer (Molecular Devices LCC, Sunnyvale, CA). The ATP standard solution concentrations and corresponding signal intensities were plotted, and the plot was extrapolated to the x-intercept. The sample ATP concentration is quantified as the absolute value of the x-axis intercept.

In addition, control and T1D RBC samples were prepared to compare the ATP release in different conditions. 7% RBC samples were prepared with either 20 nM Zn^2+^ and 20 nM C-peptide or no Zn^2+^ and C-peptide in the presence or absence of albumin. Two sample sets were created (control and T1D) with the four conditions. RBC-derived ATP release was determined in the same manner as above.

## Supplementary information

Supplementary file 1
