# Supplementary material for: A C-peptide complex with albumin and Zn2+ increases measurable GLUT1 levels in membranes of human red blood cells
Source: Sci Rep. 2020 Oct 15;10:17493. doi: 10.1038/s41598-020-74527-6 (PMC7566639; doi:10.1038/s41598-020-74527-6)
Supplement: Supplementary file 1 [file 41598_2020_74527_MOESM1_ESM.docx]

A Molecular-Level Reappraisal of a C-peptide Complex with Albumin and Zn^2+^ on Membranes of Human Red Blood Cells Supplementary Information

Geiger, M.;^2,3^Janes, T.;^2,3^ Keshavarz, H.;^2,3^ Summers, S.; ^1,3^ Pinger, C.;^1, 3^ Fletcher, D.;^4^ Zinn, K.;^1,3^ Tennakoon, M.;^5^ Karunarathne, A.;^5^ Spence, D.^1,3*^

^1^Department of Biomedical Engineering

^2^Department of Chemistry

^3^Institute for Quantitative Health Sciences & Engineering

Michigan State University

East Lansing, MI, 48824

^4^Sparrow Hospital

East Lansing, MI 48823

^5^Department of Chemistry and Biochemistry

The University of Toledo

Toledo, OH 43606

[spenceda@msu.edu](mailto:spenceda@msu.edu)

517.353.1116

**Binding of C-peptide to Radiolabeled Albumin**

In order to determine that C-peptide binds to BSA-^99m^Tc in a similar manner as unlabeled BSA, an ultrafiltration separation method was utilized. Ultrafiltration cups were 3D-printed using a Stratasys J750 3D-printer (Stratasys, Eden Prairie, MN) to fit inside of a 1.7 mL centrifuge tube and 20,000 MWCO membranes (Thermo Scientific) were cut to fit inside of the cups. Samples were made to contain either 2,700 nM BSA-^99m^Tc and 20 nM C‑peptide or 2,700 nM BSA and 20 nM C-peptide. In addition, 20 nM C-peptide controls were made without BSA. For each sample, 200 μL of solution was added to separate filtration devices and centrifuged at 10,000 xg for 1 hour. The filtered solution was diluted for C-peptide ELISA (ALPCO) to quantify the concentration of free C-peptide. The free C‑peptide concentration was then subtracted from the total control to determine the concentration of bound C-peptide.

**Supplemental Figure 1: Ultrafiltration Experiment.** This experiment, which essentially is a rapid equilibrium dialysis determination, confirmed that BSA-^99m^Tc carried C‑peptide (18.8 (±0.3) nM C‑peptide, with nK_a_=4.56 (±1.04) X 10^6^) in a similar manner as unlabeled BSA (BSA bound 19.3 (±0.2) nM with nK_a_= 6.25 (±1.08) X 10^6^) (n=6, error=SEM, #p>0.1).

#

**Supplementary Figure 2:** **Albumin binding to RBCs.** A. A saturation experiment conducted to determine the specific binding of BSA to RBC. This was completed by preparing two sample types of BSA-^99m^Tc with RBCs and subtracting the resulting data. One sample set was prepared in AF-PSS to represent total binding (black) and the other sample set was prepared in PSS so the excess unlabeled BSA could act as a blocking agent to demonstrate non-specific binding (grey) (n=6, error=SEM). B. A repeated saturation experiment of BSA specific binding to RBCs in the presence of C-peptide and Zn^2+^ (n≥4, error=SEM).


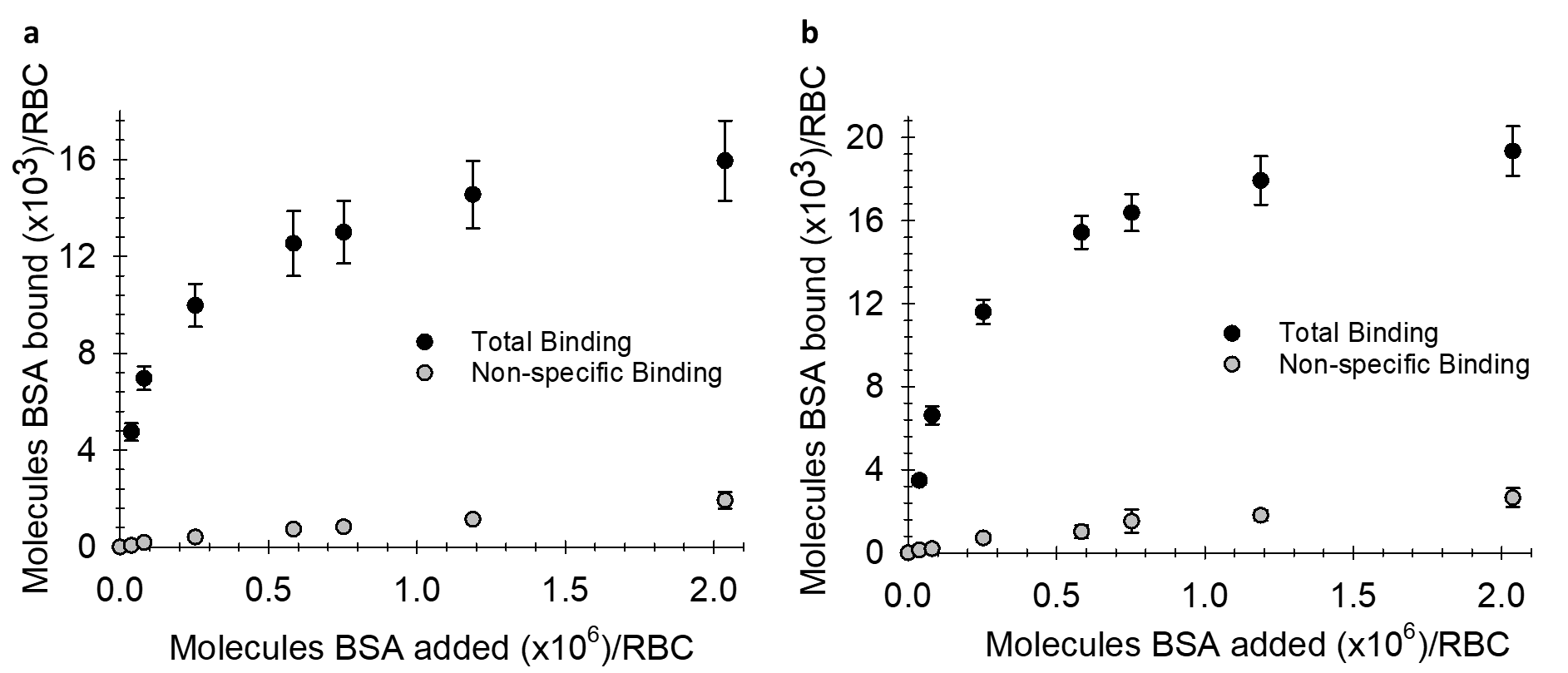


**Radioactive ^65^Zn^2+^ binding determination**

To determine Zn^2+^ binding to RBCs, the β-decay of ^65^Zn^2+^ was detected. Samples were prepared with 7% RBCs, varying concentrations of ^65^Zn^2+^ (Perkin Elmer), or a combination of C-peptide and ^65^Zn^2+^ (1, 2.5, 5, 10, 20, 50 nM) in PSS. Samples were incubated for 2 hours at 37 °C before being centrifuged at 500 xg for 5 minutes. For detection, 200 μL of the sample supernatant was mixed with 100 μL of Ultima Gold Scintillation Cocktail (Perkin Elmer) in a 96-well plate and analyzed using a MicroBeta scintillation counter (Perkin Elmer). The supernatant of each sample was analyzed in this way, and the amount of ^65^Zn^2+^ binding was determined by subtracting the remaining ^65^Zn^2+^ concentration from the initial amount added.

**Supplementary Figure 3: RBC Zn^2+^ binding.** A. RBC ^65^Zn^2+^ binding in the presence and absence of C-peptide as increasing concentrations of ^65^Zn^2+^ and C-peptide are added to the RBCs (along the x-axis). The closed circles represent ^65^Zn^2+^ alone and the open circles represent ^65^Zn^2+^ and C-peptide in the presence of albumin. B. RBC ^65^Zn^2+^ binding with C‑peptide in the presence of albumin shown on a smaller scale to more clearly highlight the specific binding curve measured (n≥8, error=SEM, *p<0.05).


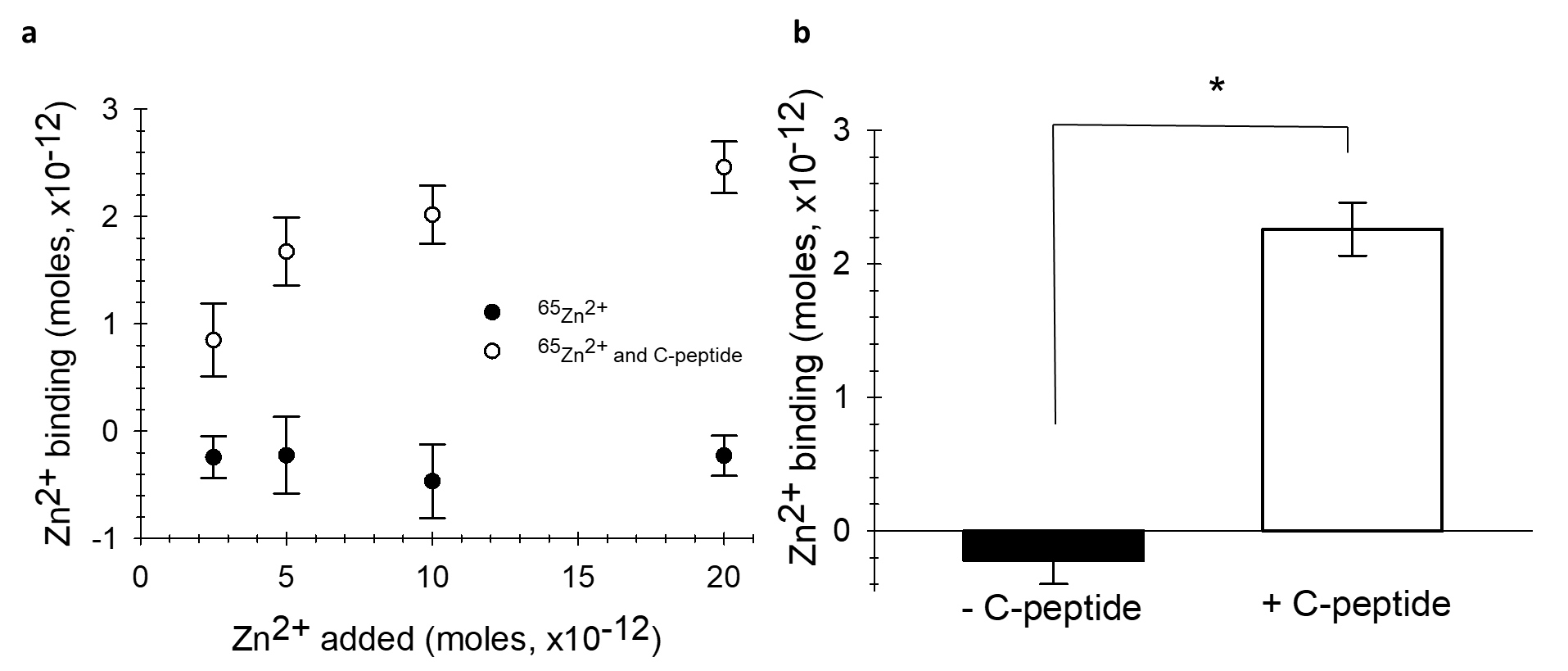


**Supplementary Figure 4: Resulting Western Blots.** Resulting β-1 spectrin and GLUT1 bands from varying solution combinations. Six samples were prepared with increasing C‑peptide concentrations and an additional six samples were prepared with increasing Zn^2+^ concentrations, both with albumin (concentrations of 0, 2.5, 5, 10, 20, and 50 nM from left to right). Then six samples were made with the same increasing concentrations of C-peptide and Zn^2+^ with albumin. After normalizing the GLUT1 bands to β‑1 spectrin, a steady increase is only detected when all three components of the complex are present. Actin was previously tried as a housekeeping protein, but the bands overlapped with those of GLUT1.


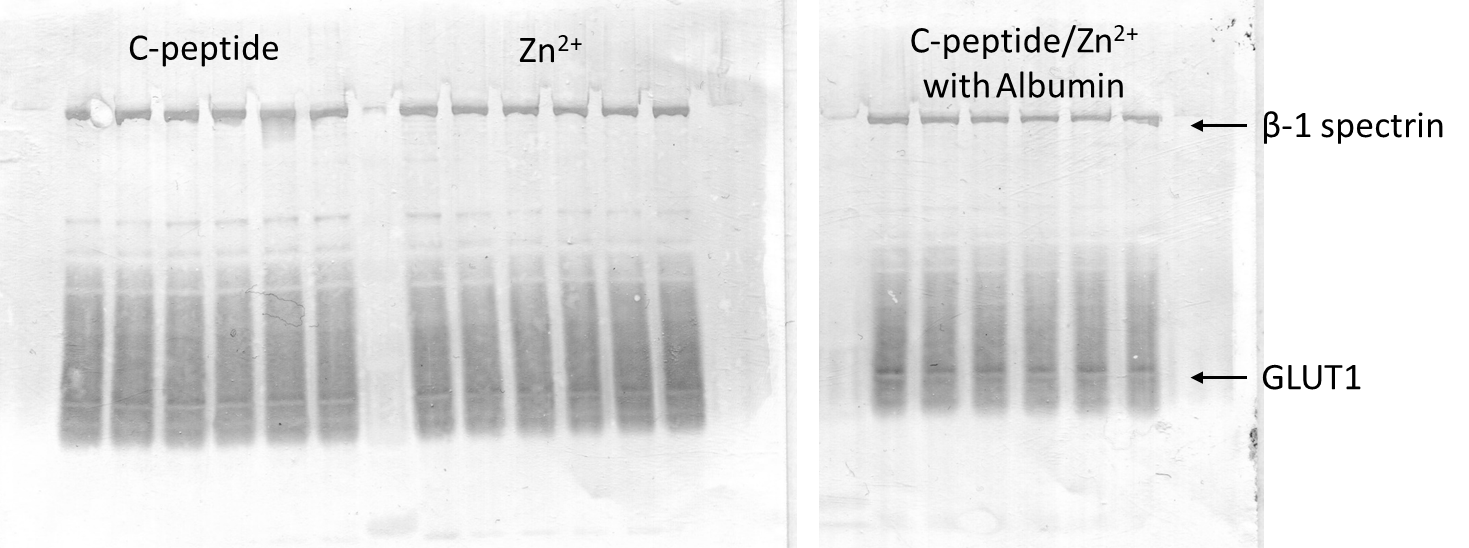

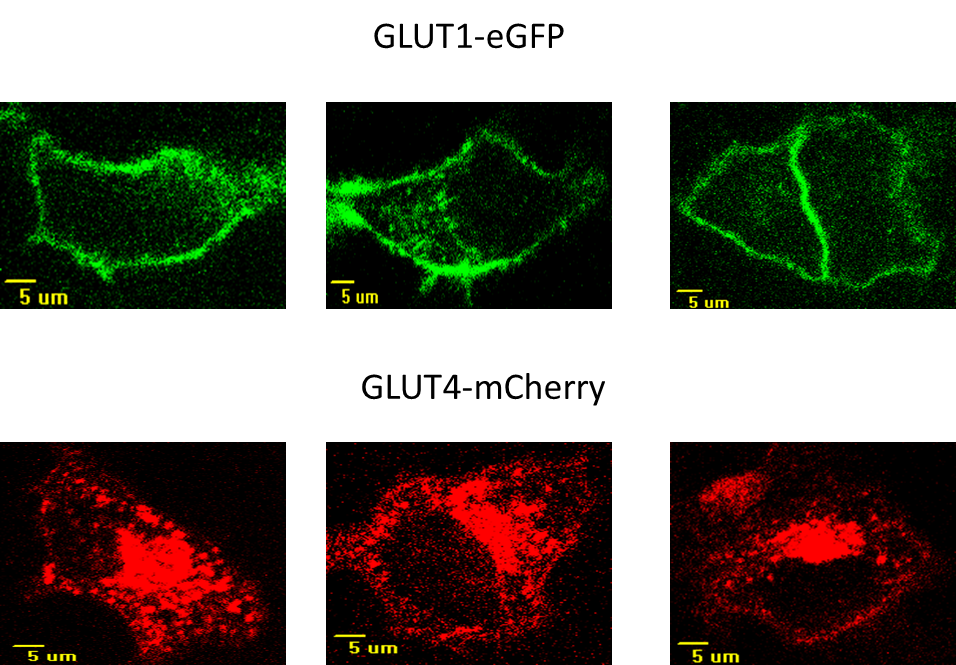


**Supplementary Figure 5: GLUT1 Clustering on CHO cells.** The clustering of GLUT1 (labeled with eGFP) and GLUT4 (labeled with mCherry) on CHO cells prior to the addition of albumin, C-peptide, and Zn^2+^.


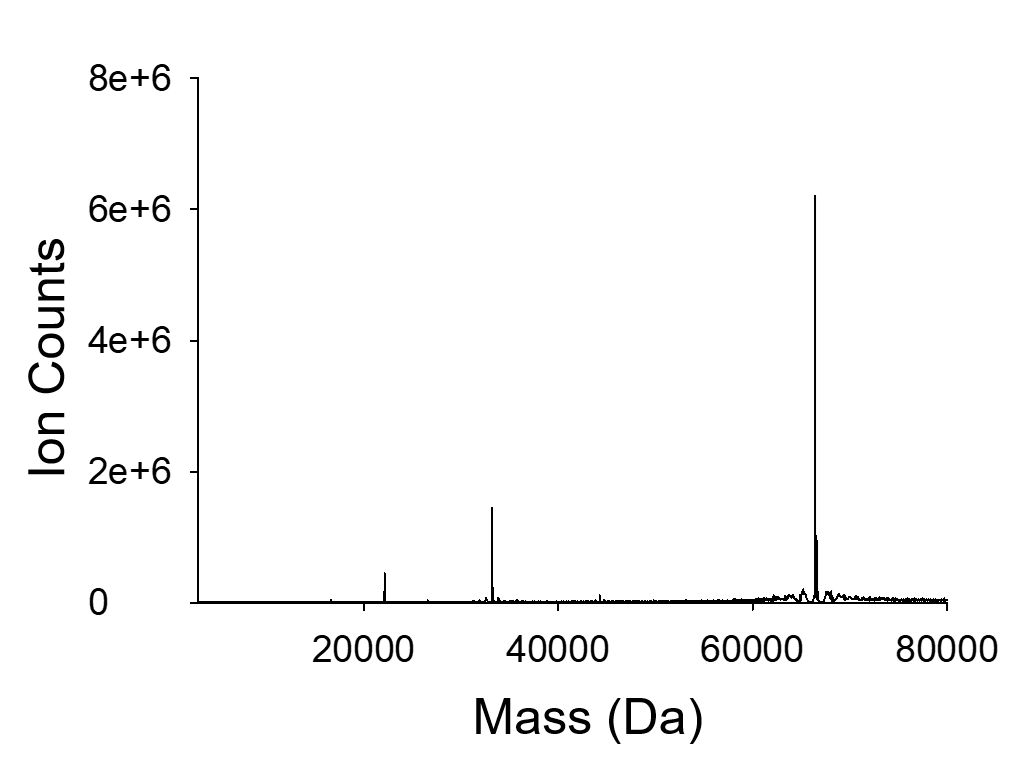


**Supplementary Figure 6: Bovine Serum Albumin Mass Spectrum.** The resulting mass spectrum from bovine serum albumin (BSA) purchased from Sigma Aldrich. Peaks at 22kDa, 33 kDa and 66.5 kDa are BSA at varying charged states. Based on the total ion counts, the commercially purchased BSA purity is ≥ 99% with no apparent other large molecules capable of carrying the C-peptide.
